# Supplementary material for: Cloud BioLinux: pre-configured and on-demand bioinformatics computing for the genomics community
Source: BMC Bioinformatics. 2012 Mar 19;13:42. doi: 10.1186/1471-2105-13-42 (PMC3372431; doi:10.1186/1471-2105-13-42)
Supplement: Additional file 1 — Supplementary 1 Cloud BioLinux software documentation in the form of a mini, self-contained website. Users need to download and uncompress the .zip file, and open through a web browser the "index.html" file available on the main directory. (ZIP 1823 kb). [file 1471-2105-13-42-S1.ZIP › Cloud-BioLinux-Package-Documentation/docs/seaview.html]

Bio-Linux Software Documentation Pages

Back to search form

## seaview

|  |  |
| --- | --- |
| Name | seaview |
| Description | **SeaView** is a graphical multiple sequence alignment editor developped by Manolo Gouy.  **SeaView** can be used as a standalone application or as a helper application when using the World Wide Web. In this case, prospective users must configure their WWW browser in a way to make it able to recognize the MIME-type associated to the data read by SeaView: chemical/mase.  **References:**  Galtier, N., Gouy, M. and Gautier, C. (1996) SeaView and Phylo\_win, two graphic tools for sequence alignment and molecular phylogeny. Comput. Applic. Biosci., 12, 543-548.[Entrez] |
| Homepage | http://pbil.univ-lyon1.fr/software/seaview.html |
| Remote Documentation | ftp://pbil.univ-lyon1.fr/pub/mol\_phylogeny/seaview/seaview.help |
